# The E3 ubiquitin ligase SMURF1 regulates cell-fate specification and outflow tract septation during mammalian heart development

**DOI:** 10.1038/s41598-018-27854-8

**Published:** 2018-06-22

**Authors:** K. Koefoed, J. Skat-Rørdam, P. Andersen, C. B. Warzecha, M. Pye, T. A. Andersen, K. D. Ajbro, E. Bendsen, M. Narimatsu, F. Vilhardt, L. B. Pedersen, J. L. Wrana, R. H. Anderson, K. Møllgård, S. T. Christensen, L. A. Larsen

**Affiliations:** 1 Department of Cellular and Molecular Medicine, University of Copenhagen, Copenhagen, Denmark; 2 Division of Cardiology, Department of Medicine, Johns Hopkins University School of Medicine, Baltimore, USA; 3 Department of Biology, University of Copenhagen, Copenhagen, Denmark; 4 Samuel Lunenfeld Research Institute, Mount Sinai Hospital, Toronto, Canada; 5 Fertility Clinic, Department of Obstetrics and Gynaecology, University Hospital of Odense, Odense, Denmark; 6 Institute of Genetic Medicine, Newcastle University, Newcastle upon Tyne, United Kingdom

## Abstract

Smad ubiquitin regulatory factor 1 (SMURF1) is a HECT-type E3 ubiquitin ligase that plays a critical role in vertebrate development by regulating planar cell polarity (PCP) signaling and convergent extension (CE). Here we show that SMURF1 is involved in mammalian heart development. We find that SMURF1 is highly expressed in outflow tract cushion mesenchyme and *Smurf1*^−/−^ mouse embryos show delayed outflow tract septation. SMURF1 is expressed in smooth muscle cells of the coronary arteries and great vessels. Thickness of the aortic smooth muscle cell layer is reduced in *Smurf1*^−/−^ mouse embryos. We show that SMURF1 is a negative regulator of cardiomyogenesis and a positive regulator of smooth muscle cell and cardiac fibroblast differentiation, indicating that SMURF1 is important for cell-type specification during heart development. Finally, we provide evidence that SMURF1 localizes at the primary cilium where it may regulate bone morphogenetic protein (BMP) signaling, which controls the initial phase of cardiomyocyte differentiation. In summary, our results demonstrate that SMURF1 is a critical regulator of outflow tract septation and cell-type specification during heart development, and that these effects may in part be mediated via control of cilium-associated BMP signaling.

## Introduction

Heart development is a complex process involving different cell types as well as different types of cell movements. At least three different cell sources contribute to heart development, including cells from first and secondary heart field (FHF and SHF, respectively), cardiac neural crest cells (CNC) and cells from the proepicardial organ (PEO). The process of endothelial to mesenchymal transition (EndoMT) is important for cardiac septation in the atrioventricular canal (AVC) and outflow tract (OFT). In the mouse, at the onset of OFT septation (E10.5), the cardiac tube consists of an outer myocardial and an inner endocardial layer separated by a layer of extracellular matrix (cardiac jelly)^1,2^. As endocardium-derived mesenchymal cells begin to invade the cardiac jelly by EndoMT in the proximal OFT cushions, CNC cells de-laminate by convergent extension (CE) to migrate through the pharyngeal arches to populate the distal part of the OFT to participate in formation of the aortopulmonary septum, and also enter the major outflow cushions^3–7^. By E12.5, the aortopulmonary septum has fused with the distal end of the outflow cushions, and septation of the OFT is completed^8^.

SMURF1 is a HECT-type E3 ubiquitin ligase, which predominantly patterns ectodermal tissues. Aberrant expression of the protein results in ectodermal-derived defects such as neural tube closure defects^9–11^. Mice with loss of *Smurf1* expression are vital and fertile with no apparent developmental defects^12^. Double knockout mouse embryos lacking SMURF1 and the homologous protein, SMURF2, however, die prenatally around E10.5. They present with neural tube closure defects, reminiscent of phenotypes seen in morphant *Smurf1 Xenopus* and zebrafish embryos^9,11,13,14^. Loss of three of four *Smurf* alleles in mice, with one *Smurf1* allele remaining, results in defects similar to the *Smurf1/*2 double knockout mice, although the mice survive gestation^13^. This suggests that there is some degree of redundancy between SMURF1 and SMURF2, which correlates well with the proteins being co-expressed in e.g. pharyngeal arches and ectodermal structures in *Xenopus* embryos^15^. The phenotypes observed in *Smurf1/2* double knockout mice suggest that SMURF proteins are involved in regulation of planar cell polarity (PCP) signaling and CE during development^13^. In support of a role in regulating CE, previous studies indicated a role for SMURF1 in regulating cell polarity, cell migration and EMT through local ubiquitination of the small GTPase RHOA at cellular protrusions^16–19^. Several studies have pointed to a role of SMURF proteins in cardiovascular development. For example, *Smurf1 and 2* are highly expressed in the mouse embryonic heart^13^. SMURF1 is involved in EndoMT processes in chicken AVC explants and in mouse epicardial cells^18,20^. Previously, a 480 kbp *de novo* duplication including *SMURF1* was identified in a screen for copy number variants in a cohort of patient with congenital heart defects (CHD)^21^ and a *de novo* frameshift mutation in *SMURF1* was recently associated with left-sided CHD^22^. The precise function of SMURF proteins in heart development, nonetheless, remains poorly understood.

At the molecular level, SMURF proteins have been implicated in the positive and negative regulation of numerous cellular and developmentally important signaling pathways, including canonical TGFβ/BMP signaling as well as WNT/PCP signaling, TGFβ/PAR6/RHOA, Hedgehog, Hippo and NF-κB signaling^9,12,13,16–19,23–27^. The majority of these pathways are known to be coordinated, at least in part, by the primary cilium - a microtubule-based signaling organelle that emerges from the surface of many different cell types in the body depending on their cell cycle and differentiation status^28–34^. In this context, it is noteworthy that SMURF1 and 2 were shown recently to promote activation of Sonic hedgehog (Shh) signaling by mediating the ubiquitination and endocytic clearance of the Shh receptor Patched1 from the ciliary compartment^24^. In addition, SMURF1 was reported to function as a negative regulator of TGFβ/BMP signaling in developing *Xenopus* embryos by targeting SMAD transcription factors and receptors for degradation^9,23,35,36^. SMAD-mediated TGFβ/BMP signaling has also shown to be associated with the primary cilium^37–41^, for example during differentiation of mouse carcinoma stem cells (P19.CL6 cells) into cardiomyocytes where TGFβ-mediated phosphorylation of SMAD2/3 at the ciliary base is required for the process of cardiomyogenesis^40^. Despite these findings, the potential link between SMURF proteins and the primary cilium remains unclear.

In this study, we used human embryonic hearts, as well as wild type and mutant mouse embryos and stem cell models, to address the role of SMURF1 during heart development, and to examine the mechanisms involved. Using these approaches, we demonstrate that SMURF1 regulates OFT septation and cell-type specification during heart development by a mechanism that may involve SMURF1-mediated regulation of cilium-associated BMP signaling. These results provide important new insight into the process of OFT septation and the mechanisms that define cell-type specifications during cardiac development, in turn paving the way for improved *in vitro* differentiation of cardiomyocyte subtypes for use in treatment of cardiovascular diseases.

## Results

### SMURF1 is expressed in a spatiotemporal manner during human heart development

**Figure 1:**
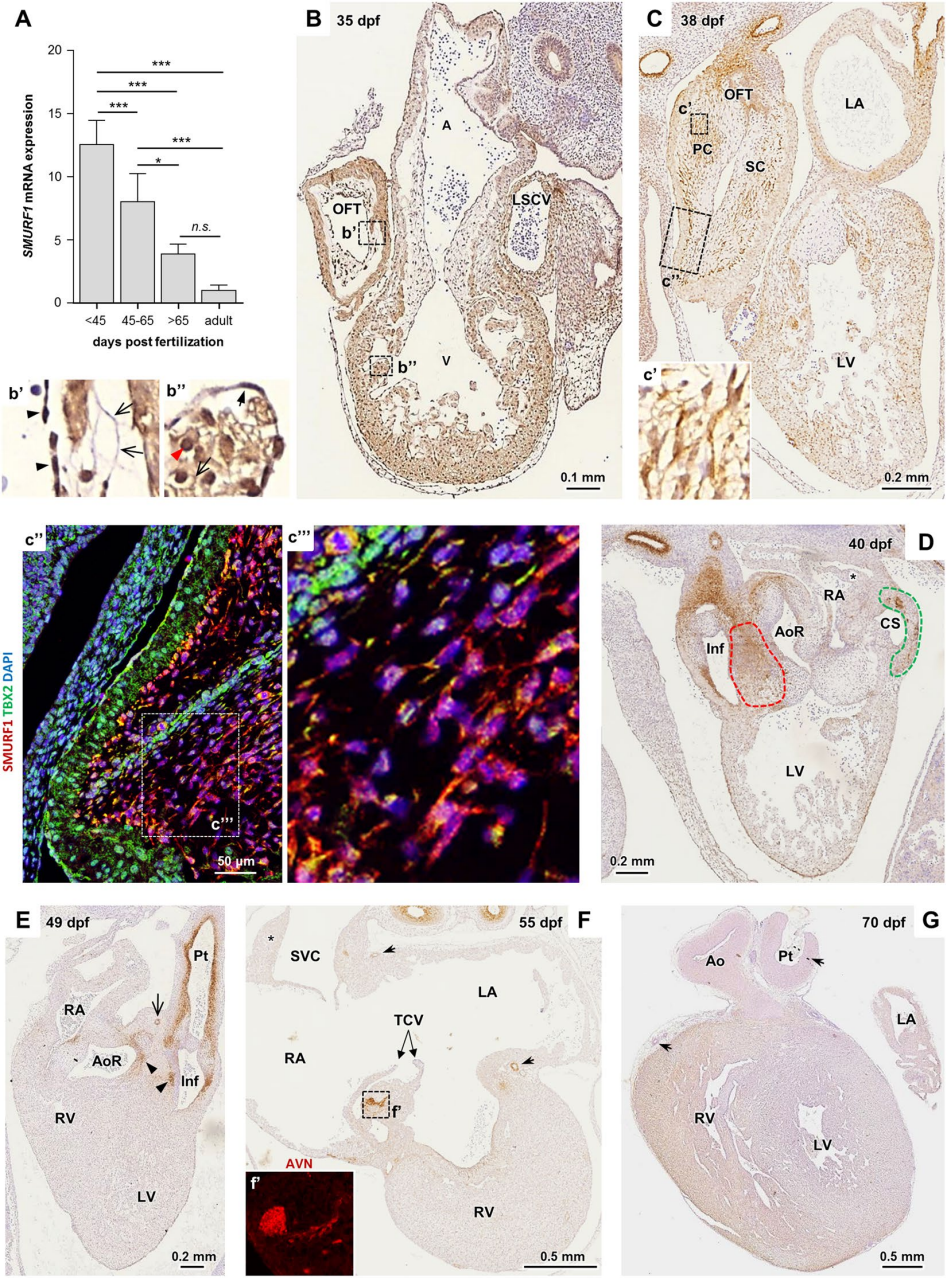
Expression of SMURF1 during human heart development. (**A**) *SMURF1* expression in human embryonic, fetal and adult hearts. Expression level was examined using qRT-PCR and normalized to average values of three housekeeping genes (*GAPDH*, *COX4* and *ATP6*). Expression level is shown as fold change compared to expression in the adult heart. Variations between groups were compared using a one-way ANOVA. *p < 0.05, ***p < 0.001, ****p < 0.0001. (**B**) IHC staining of SMURF1 in a human heart section from 35 dpf. b’ and b” enlargement of boxes indicated in B; closed arrow heads show endothelial cells, open arrow heads show membrane localization and red arrow shows nuclear localization (see Supplemental Fig. 1 for enlargement). (**C**) IHC staining of SMURF1 in a human embryonic heart section from 38 dpf. c’ Enlargement of the boxed area in C. c” IF-IHC image of a neighboring section in the area marked by the box in C (SMURF1 (*red*), TBX2 (*green*); nuclei are stained with DAPI (*blue*). c”’ enlargement of box indicated in c”. (**D**) IHC staining of SMURF1 in a human embryonic heart from 40 dpf. Red outline: condensed CNC mesenchyme, green outline: patches of SMURF1 positive cells in the coronary sinus. Asterisk indicates the primary atrial septum. (**E**) SMURF1 staining in human embryonic heart from 49 dpf. Closed arrow heads show remnants of condensed CNC-derived mesenchyme, open arrow head marks the coronary vessel. (**F**) SMURF1 staining in human embryonic heart from 55 dpf. Asterisk marks area of sinus atrial node and open arrows mark coronary vessels. f’ IF-IHC of SMURF1 in a neighboring section showing SMURF1 expression (*red*) in an the boxed area in F. (**G**) SMURF1 staining in human embryonic heart from 70 dpf. Arrows mark SMC-positive areas. Abbreviations: A: Atrium, Ao: Aorta, AoR: Aortic root, AVN: Atrioventricular node, CS: Coronary sinus, Inf: Infundibulum, LA: Left atrium, LSCV: Left superior caval vein, LV: Left ventricle, OFT: Outflow tract, PC: Parietal cushion, Pt: Pulmonary trunk, RA: right atrium, RV: Right ventricle, SC: Septal cushion, SVC: Superior vena cava, TCV: Tricuspid valves, V: Ventricle.

To investigate the expression pattern of SMURF1 during human heart development, we first analyzed the relative *SMURF1* mRNA levels of 20 human embryonic hearts, ranging from 39–68 days post fertilization (dpf), as well as three adult hearts, by quantitative reverse transcriptase (qRT)-PCR. This analysis showed that *SMURF1* expression is about 12-fold higher in 39–44 dpf embryonic hearts compared to adult hearts (Fig. 1A). Next, we examined the spatial expression pattern of SMURF1 in similar samples using immunohistochemistry (IHC). In 35–38 dpf embryonic hearts, SMURF1 is expressed in the myocardium and OFT cushions, with a particular strong expression in the latter (Fig. 1B and C). We also observed a variation in the subcellular localization of SMURF1 in different cell types, with SMURF1 predominantly localizing to the nucleus in cells in the endocardium of the ventricle and OFT (closed arrowheads in Fig. 1b’). Cardiomyocytes of the ventricle and atria, in contrast, display SMURF1 localization also in the cytoplasm (open arrowheads Fig. 1b”). In the myocardium of the OFT, the localization appears equally intense in the cytoplasm and nuclei (red arrowhead). At 35 dpf, SMURF1 is localized preferentially at the plasma membrane in the mesenchymal cells of the OFT cushions, as well as in the AVC (open arrows in 1b’ and Suppl. Fig. 1).

The expression of SMURF1 in the AVC cushions appears to be transient, since these cushions are negative for SMURF1 at 38 dpf. By this stage, in contrast, the expression is enriched in the OFT (Fig. 1C). SMURF1 is expressed in both OFT cushions (Fig. 1C and Suppl. Fig. 2), with a more prominent expression distally. The confocal image (from a neighboring section) in Fig. 1c” shows that SMURF1 is highly expressed in the mesenchymal cells, compared to the myocardium (marked by TBX2), and that SMURF1 expression is continuously abundant at cell membranes in the cushion mesenchyme (Fig. 1c”’). As heart development progresses, the expression of SMURF1 in the myocardium is lost, becoming restricted to more confined areas (Fig. 1D–F). At 40 dpf, SMURF1 is expressed in the CNC-derived condensed mesenchyme between the developing aortic root and the infundibulum of the pulmonary trunk (red outline in Fig. 1D), suggesting that SMURF1 may be involved in CNC-mediated septation of the OFT. At the coronary sinus, SMURF1 is expressed in a pattern reminiscent of a role in specifying the endothelium of the coronary artery (green outline in 1D)^42–44^. This pattern is also evident in a neighboring section in the 38 dpf heart (Suppl. Fig. 2). At 40 dpf SMURF1 is also expressed in the infundibulum and the proximal aorta, and in the pulmonary trunk. When the heart is fully septated at 49 dpf, SMURF1 expression is maintained in the infundibulum and the pulmonary trunk, as well as in the remnants of the condensed CNC mesenchyme (arrowheads in Fig. 1E). At 49 and 55 dpf, SMURF1 also marks the smooth muscle cells (SMCs) in the developing coronary arteries (black arrow in Fig. 1E,F). Additionally, at 55 dpf the atrioventricular node (AVN) is SMURF1-positive, whereas the sinus node remains SMURF1 negative (asterisk in 1F). At 70 dpf, SMURF1 expression appears stronger in the right ventricle (RV) compared to the left ventricle. This might suggest a specific role in the RV. By this time, expression in SMCs is lost in the great vessels and coronary arteries (arrows in 1G). Together, these results suggest that SMURF1 may be involved in OFT septation, and in development of SHF-derived structures as well as the coronary arteries and AVN.

### OFT septation is delayed and the aortic SMC layer is reduced in *Smurf1*^−/−^ mouse embryos

**Figure 2:**
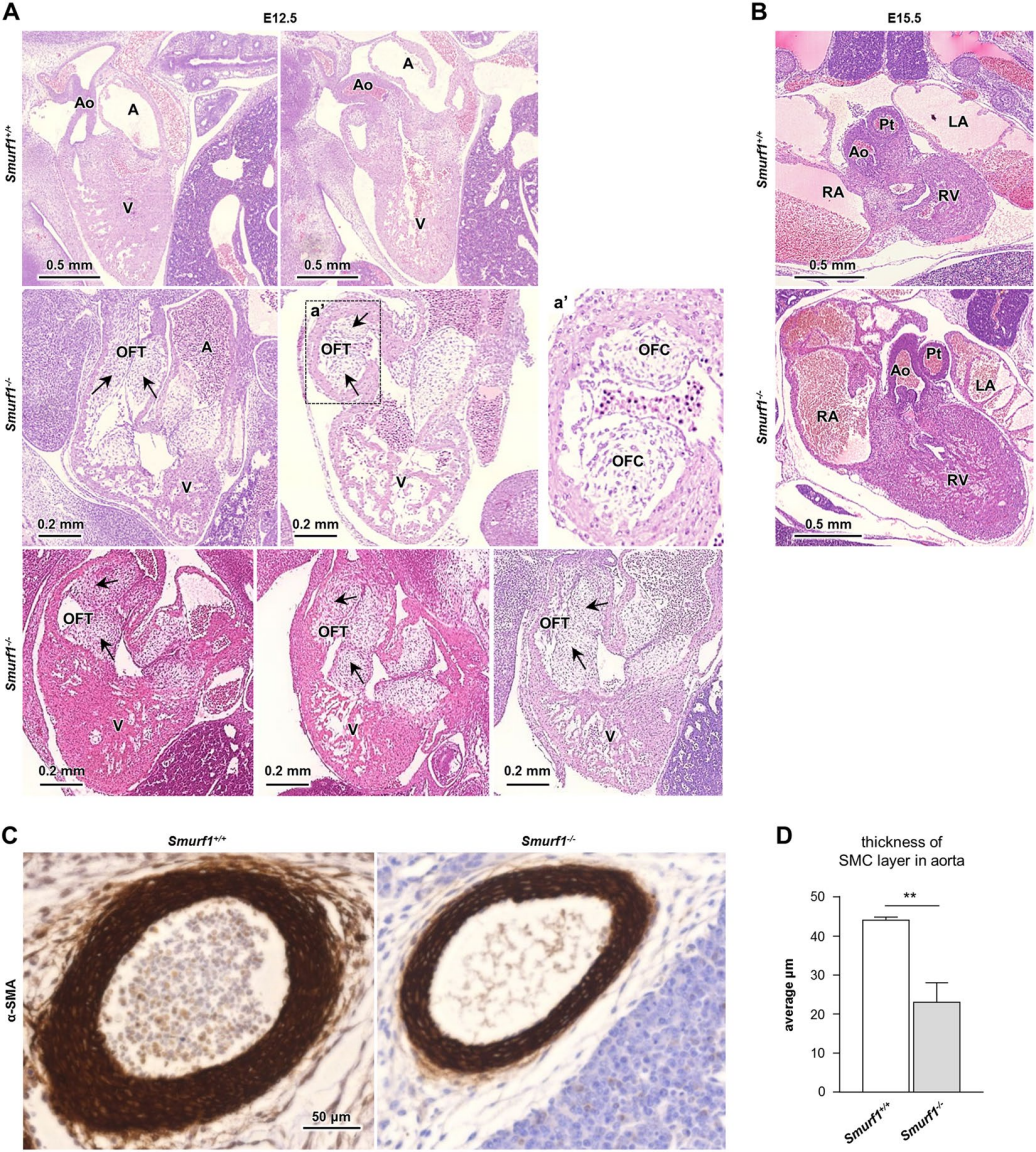
Cardiac anatomy in wildtype and *Smurf1*^−/−^ mice. (**A**) Hematoxylin stained mice hearts fixed at E12.5. Top panel shows wildtype, middle and bottom panel shows *Smurf1*^−/−^ mice. Outflow track cushions are shown with arrows. (**B**) Hematoxylin stained mice hearts fixed at E15.5. Top panel shows wildtype and bottom panel shows *Smurf1*^−/−^ mice. Abbreviations: A: Atrium, Ao: Aorta, LA: Left atrium, OFT: Outflow tract, PT: Pulmonary trunk, RA: Right atrium, RV: Right ventricle, V: Ventricle. (**C**) IHC staining of α-SMA expression in the aorta of E15.5 mice embryos from WT and *Smurf1*^−/−^ mice. The images are representative of three different WT mice and three different *Smurf1*^−/−^ mice. (**D**) Quantification of the average thickness of the SMC layer in the aorta. N = 3 (one section per mouse). An unpaired t-test was used to for statistical analysis **p < 0.01.

Mice with targeted deletion in *Smurf1* were reported to be viable with no cardiac defects^13^. Nevertheless, the strong expression of SMURF1 in the OFT in human embryonic hearts prompted us to re-examine the OFT in mouse *Smurf1*^−/−^ embryos. We analyzed hematoxylin stained tissue sections from wild type (WT) and homozygous *Smurf1*^−/−^ mice at E12.5 and E15.5. The mice were of the same background as previously published^13^. As expected, the OFT was septated at E12.5 in WT mice (n = 8) (Fig. 2A). In contrast, six out of eight *Smurf1*^−/−^ embryos presented with a common OFT at E12.5. By E15.5, both WT and *Smurf1*^−/−^ mice presented with a fully septated OFT (Fig. 2B), indicating that loss of *Smurf1* causes a delay in OFT septation. We observed strong expression of SMURF1 in SMC in the developing coronary arteries of human embryos, which might indicate a role in development of arterial tissues. This observation encouraged us to analyze the expression of the SMC marker alpha-actin-2 (α-SMA) in the aorta of *Smurf1* WT and *Smurf1*^−/−^ mice by IHC. In E15.5 mice, the thickness of the aortic SMC layer was significantly reduced in the *Smurf1*^−/−^ mice compared to the *Smurf1* WT mice (Fig. 2C,D), suggesting that SMURF1 is required for proper SMC differentiation.

### Loss of *Smurf1* increases the rate of cardiomyogenesis in P19.CL6 cells

Based on our findings that SMURF1 is expressed in the myocardium of human embryonic hearts, we next investigated the function of SMURF1 in cardiomyogenesis. We used the P19.CL6 cell line, which differentiates into cardiomyocytes upon addition of 1% DMSO to the culture medium^34,40,45^. When seeded at a density of 4,000 cells/cm^2^, the cells differentiated as expected into cardiomyocytes within eight to ten days, as judged by the formation of spontaneous contracting clusters concomitantly with a loss of *Sox2* expression and increased expression of cardiomyocyte markers *Gata4*, *Nkx2-*5 and *α-actinin* (Suppl. Fig. 3). Relative expression levels of *Smurf1* and *Smurf2* were largely constant throughout cardiomyogenesis in this assay (Suppl. Fig. 3).

**Figure 3:**
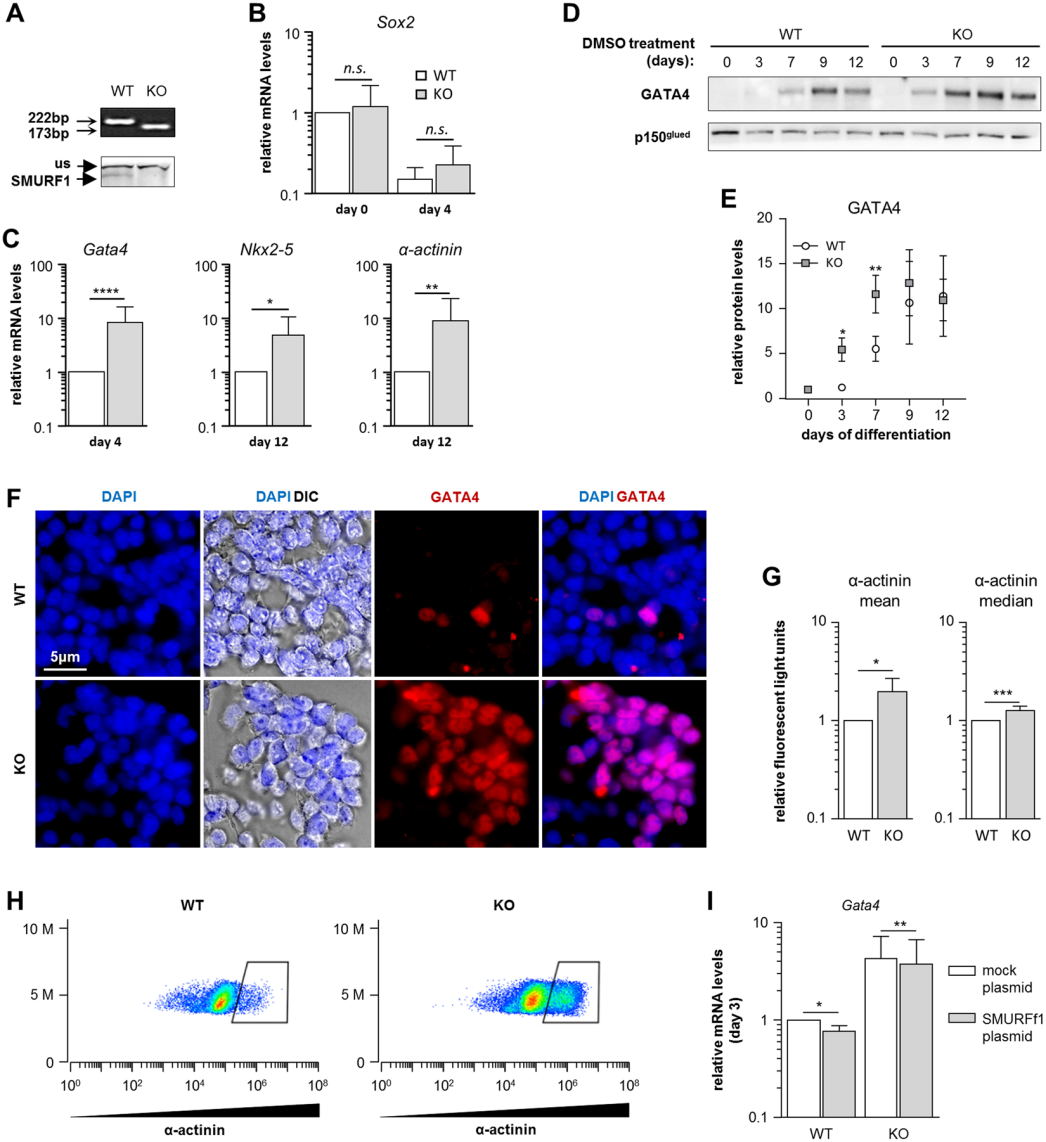
Functional analysis of SMURF1 during cardiomyogenesis in P19.CL6 cells. (**A**) Agarose gel (top panel) and WB (bottom panel) of the WT and KO clones used in the experiments. SMURF1 and unspecific (us) band in WB is marked with arrows. (**B**) qRT-PCR analysis of *Sox2* expression on before (day 0) and after DMSO stimulation (day 4) in WT and KO cells. Data are normalized to *Gapdh* and *Psmd4*. Ratio paired t-test was used for statistical analysis, n = 10. (**C**) qRT-PCR analysis of *Gata4* expression on day 4 of DMSO stimulation and *Nkx2-5* and *α-actinin* on day 12 of DMSO stimulation. Data are normalized to *Gapdh* and *Psmd4*. Ratio paired t-test was used for statistical analyzes *p < 0.05 **p < 0.01****p < 0.0001, n = 10 for *Gata4*, and n = 8 for *Nkx2-5* and *α-actinin*. (**D**) WB analysis of GATA4 expression during differentiation in WT and KO cells. p150^glued^ was used as a loading control. The WB showed is representative of three experiments. (**E**) Quantification of GATA4 protein expression during differentiation. Paired t-test was used for statistical analyses, *p < 0.05, **p < 0.01, n = 3. (**F**) Immunofluorescence and Differential interference contrast (DIC) microscopy analysis of WT and KO cells on day 3 of DMSO stimulation. GATA4 (*red*). Nuclei were stained with DAPI (*blue*). (**G**) Flow cytometry analysis of α-actinin on day 12 of DMSO stimulation in KO and WT cells. Graphs depict the mean and median of the Relative Fluorescent Light Units with α-actinin antibody. Paired t-test was used for statistical analyses *p < 0.05, ***p < 0.001, n = 6. (**H**) Density plot of a representative flow cytometry experiment with WT and KO cells. (**I**) qRT-PCR analysis of *Gata4* mRNA expression on day 3 of DMSO stimulation in WT and KO cells treated with either a Mock or *Smurf1* expressing plasmid. Data are normalized to *Gapdh* and *Psmd4*. A ratio paired t-test was used to for statistical analysis *p < 0.05, **p < 0.01. Original pictures of western blots and the agarose gel are shown in Supplemental Fig. 5.

Next, to characterize the function of SMURF1 during cardiomyogenesis in P19.CL6 cells, we used CRISPR-Cas9 genome editing to generate stable clones with a 49 bp deletion in exon three of the *Smurf1* gene (Suppl. Fig. 4A). We randomly selected three clones with wildtype *Smurf1* sequence as controls (designated WT in the following), and seven homozygous mutants (designated KO) for further analyses. All ten cell clones were tested by Sanger sequencing and western blot (WB) analysis to ensure that they were either WT or KO with the expected deletion. Figure 3A shows representative examples of results from PCR and WB analyses of a WT clone and a homozygous mutant with the top panel displaying PCR products spanning the site of mutation and the bottom panel showing a WB where the SMURF1 protein band is missing in the KO, verifying that the mutation results in depletion of SMURF1 protein. The ten isolated clones were subjected to cardiac differentiation, and the expression level of *Gata4* was analyzed at day four as a measure of differentiation stage (Suppl. Fig. 4D). The results suggest that the KO mutants display an increased rate of differentiation as compared to the WT clones. Two KO mutants (KO#1 and KO#2) and two controls (WT#A and WT#B) were chosen for further analysis (Suppl. Fig. 4E). All differentiation experiments were carried out with these four clones, and as shown in Suppl. Fig. 4F, the two WT clones displayed a similar level of *Gata4* and *α-actinin* expression at days 4 and 12 of differentiation, respectively, whereas the two KO clones display a significant increase in the expression of *Gata4* and *α-actinin* compared to the WT clones. For simplicity, the results represented in the main figures only include WT#A and KO#1, which are designated WT and KO, respectively.

In zebrafish, it has been reported that morphants behave differently than mutants because the mutants adapt to the mutation by adjusting the transcriptome^46^. Similarly, *Smurf2* was reported to be significantly upregulated in the *Smurf1*^−/−^ mouse, indicating that the mutant mice compensate by increasing *Smurf2* transcription^13^. We therefore investigated whether the KO cells had compensated for the loss of SMURF1 by up-regulating expression of *Smurf2*. However, *Smurf2* mRNA levels were not significantly different on days 0 and 4 in the KO versus WT cells, as assessed by qRT-PCR analysis (Suppl. Fig. 4G). We analyzed the relative expression *Sox2* mRNA and the cardiomyocyte markers *Gata4*, *Nkx2-*5 and *α-actinin* at days 0, 4 and 12 of differentiation to compare the differentiation dynamics between WT and KO cells. We did not observe any difference in *Sox2* expression, indicating that the cells leave their pluripotent state at a similar time point after addition of DMSO (Fig. 3B). In line with the initial analysis (Suppl. Fig. 4D), the mRNA levels of *Gata4* at day 4 and *Nkx2*.*5* and *α-actinin* at day 12 were 5–10 times higher in KO cells compared to WT cells (Fig. 3C). In support of these findings, WB analysis showed that KO cells express GATA4 at a significantly higher level than that observed in WT cells at days 3 and 7 of differentiation (Fig. 3D,E), which coincides with an increased number of cells positive for GATA4 as judged by IFM analysis (Fig. 3F). Furthermore, we analyzed the expression of α-actinin using flow cytometry in order to compare the fraction of cells differentiated into functional cardiomyocytes at day 12. Both the mean and the median of relative fluorescent light units was higher in the KO culture compared to the WT culture (Fig. 3G), reflecting that a significantly larger proportion of cells in the KO culture expresses a high level of α-actinin compared to the WT culture. This is also evident from the density plots displayed in Fig. 3H. We transfected both WT and KO cells with either a mock or a *Smurf*1-expressing plasmid to see if we could rescue the phenotype. As shown in Fig. 3I, introduction of a transient increase in *Smurf*1 expression in the KO cells at day 3 of differentiation resulted in a slight but significant decrease in *Gata4* expression. The same tendency was seen when *Smurf*1 was transiently over-expressed in WT cells (Fig. 3I). Taken together, our results suggest that SMURF1 inhibits cardiomyogenesis in P19.CL6 cells.

### Depletion of *Smurf1* affects the formation of mESC-derived SHF cells

**Figure 4:**
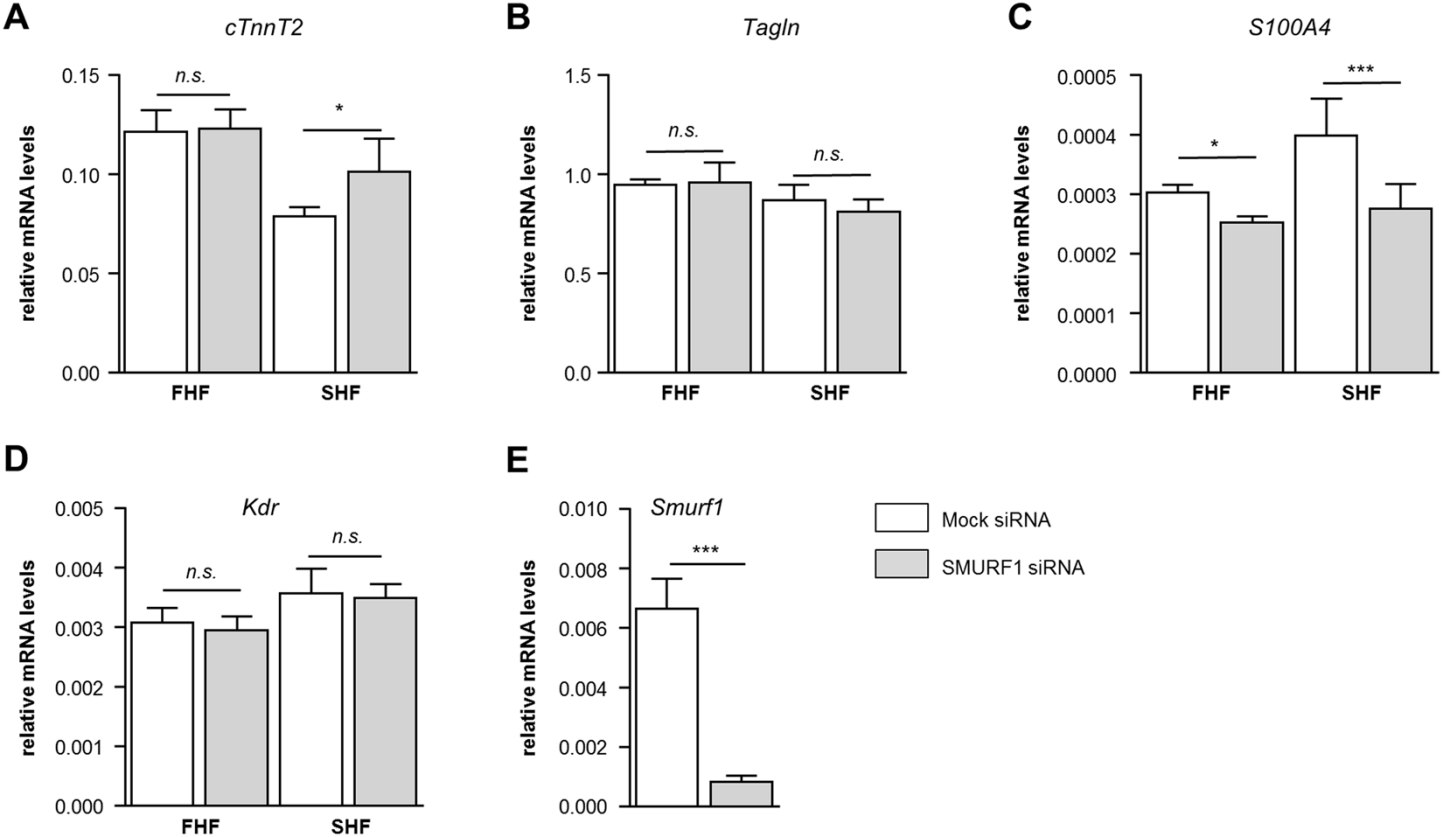
Functional analysis of SMUR1 during cardiomyogenesis in mESCs. qRT-PCR of the relative expression of (**A**) *TnnT2*, (**B**) *Tagln*, (**C**) *S100A4* and (**D**) *Kdr* in FHF and SHF cells with or without transient knockdown of *Smurf1* n = 4 (**E**) qRT-PCR of the relative expression of *Smurf1* in mixed FHF and SHF population 24 hours after transfection.

The role of SMURF1 as a negative regulator of cardiomyogenesis was further investigated in mouse embryonic stem cells (mESC). We used a mESC line which expresses GFP and RFP when the cells differentiate into FHF (GFP+) and SHF (RFP+) derivatives, respectively^47^. These cells were analyzed for their ability to differentiate into cardiomyocytes, vascular endothelial cells (VEC), SMC cells and cardiac fibroblasts upon siRNA induced *Smurf1* depletion. After an initial differentiation period of five days the RFP+ and GFP+ cells were FACS-sorted into two pools. Cells were treated with siRNA against *Smurf1* and allowed to differentiate for additional 72 hours followed by RNA isolation and qRT-PCR analysis. The cells were analyzed for expression of the cardiomyocyte marker cardiac muscle troponin T (*TnnT*2), the SMC marker Transgelin (*Tagln*), the VEC marker kinase insert domain receptor (*Kdr*), and the cardiac fibroblast marker S100 calcium-binding protein A4 (*S*1*00A4*) (Fig. 4). The expression of *TnnT2* was not affected by *Smurf1* knockdown in FHF cells, however in SHF cells *Smurf1* depletion resulted in a significant increase of *TnnT2* expression (Fig. 4A) reminiscent of what we observed in P19.CL6 cells. Interestingly, the increase in *TnnT2* expression in SHF cells seems to be on behalf of cardiac fibroblasts (Fig. 4C). In FHF cells, there is also a significant reduction of cardiac fibroblasts, suggesting that SMURF1 in general is a positive regulator of cardiac fibroblasts. The fact that a phenotype is only observed in SHF cells suggests that SMURF1 may regulate cell-type specification during cardiomyogenesis in SHF derived structures of the heart. Further, the reduced expression of *S100A4* suggests that SMURF1 may be a positive regulator of cardiac fibroblasts.

### SMURF1 localizes to the primary cilium and regulates BMP signaling *in vitro* and *in vivo*

**Figure 5:**
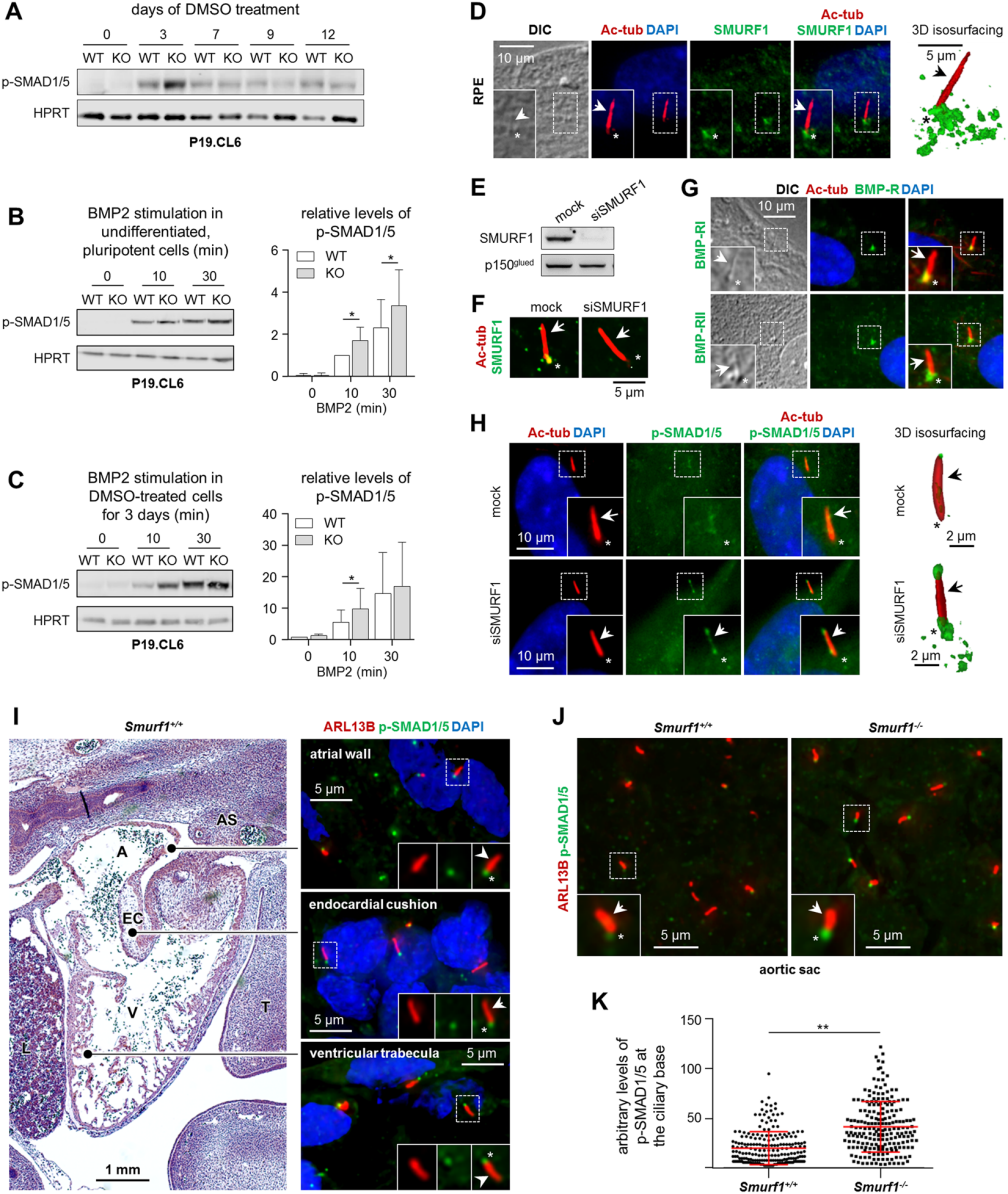
SMURF1 regulates BMP signaling. (**A**) Representative image of SDS-PAGE and WB analysis of the level of phospho-SMAD1/5 (p-SMAD1/5) during differentiation in WT and KO P19.CL6 cells (n = 3). (**B**) Left panel: SDS-PAGE and WB analysis of p-SMAD1/5 in P19.CL6 cells differentiated to day three and stimulated with 200 ng/ml BMP2. Right panel: Quantification of the relative levels of p-SMAD1/5 presented in the left panel (n = 5). Paired t-test was used for statistical analyses *p < 0.05. (**C**) Left panel: SDS-PAGE and WB analysis of p-SMAD1/5 in un-differentiated P19.CL6 cells stimulated with 200 ng/ml BMP2. Right panel: Quantification of the relative levels of p-SMAD1/5 presented in the left panel (n = 3). Paired t-test was used for statistical analyses *p < 0.05. (**D**) Representative images from IFM analysis with 3D reconstruction and isosurface visualization on the localization of SMURF1 (*green*) to the primary cilium marked with anti-acetylated α-tubulin (Ac-tub, *red*, arrows) in RPE cells. Nuclei were stained with DAPI (*blue*). Asterisks mark the ciliary base area. (**E**) Representative images from IFM analysis on the localization of BMP receptors I and II (BMP-RI and BMP-RII, respectively) (*green*) to primary cilia (*red*) in RPE cells. (**F**) SDS-PAGE and WB analysis on the efficiency of siRNA-mediated knock-down of SMURF1 (siSMURF1) in RPE cells. (**G**) IFM analysis confirming that SMURF1 is depleted from cells subjected to siSMURF1 and that antibody recognition of SMURF1 (*green*) at primary cilium (*red*) is specific. (**H**) Representative images from IFM analysis with 3D reconstruction and isosurface visualization of the level of p-SMAD1/5 (*green*) at the primary cilium (*red*) in Mock- and siSMURF1-treated RPE cells. p150^glued^ and HPRT were used as loading controls. Original pictures of western blots are shown in Supplemental Fig. 5. (**I**) Representative images from IFM analysis of WT mouse embryonic hearts at E12.5. Phosphorylation of SMAD1/5 (p-SMAD1/5, *green*) at the base of the primary cilium (ARL13B, *red*) was observed in the atrial wall, ventricular trabeculae and endocardial cushions (EC). (**J**) Representative images of the level of p-SMAD1/5 (*green*) at the base of the primary cilium (*red*) in cells surrounding the region of the pharyngeal arch arteries in WT and *Smurf1*^−/−^ embryos. (**K**) Quantification of the arbitrary levels of p-SMAD1/5 at the ciliary base (n = 3). Paired t-test was used for statistical analyses **p < 0.01.

Multiple signaling pathways known to be regulated by SMURF1, play a critical role in heart development, including TGFβ/BMP signaling^34^ which previously was shown to be coordinated by the primary cilium to promote the differentiation of P19.CL6 cells into cardiomyocytes^33,40^. To address the role of SMURF1 in regulating BMP signaling during cardiomyogenesis, we initially monitored the level of SMAD1/5 phosphorylation during cardiomyogenesis in WT and KO P19.CL6 cells by WB analysis. As shown in Fig. 5A, the level of phosphorylated SMAD1/5 (p-SMAD1/5) is increased from day 3 of differentiation, which may indicate that SMAD1/5 activation is required for cardiomyogenesis. Indeed, the level of p-SMAD1/5 at day 3 in KO cells is significantly elevated as compared to WT cells, which is in line with the conclusion that SMURF1 negatively regulates SMAD1/5 signaling in P19.CL6 cells and when depleted from the cells enhances this signaling to increase the rate of cardiomyogenesis. Since SMAD1/5 is activated by ligands of the BMP family, we next evaluated the effect of BMP2 stimulation on p-SMAD1/5 in both pluripotent cells and in cells subjected to cardiac lineage commitment by DMSO treatment. In both cases, BMP2-mediated phosphorylation of SMAD1/5 was significantly elevated in KO cells compared to WT cells after 10 minutes of stimulation (Fig. 5B,C) as well as in undifferentiated KO cells after 30 minutes (Fig. 5B). These results support the conclusion that SMURF1 negatively regulates BMP2-mediated activation of SMAD1/5 during cardiomyogenesis.

To further evaluate the potential role of SMURF1 in regulating BMP signaling at the primary cilium, we initially performed IFM analysis on the localization of SMURF1 and BMP signaling components in cultures of RPE cells, which represent a well-established cell model system for ciliary signaling^48–50^. We found that both SMURF1 and the BMP Receptors BMP-RI and BMP-RII localize at the primary cilium in these cells (Fig. 5D,E), and that siRNA-mediated knock-down of SMURF1 (Fig. 5F,G) is associated with an increased level of p-SMAD1/5 at the cilium in un-stimulated cells (Fig. 5H). These results support the conclusion that the primary cilium contains the complement of signaling modules required for regulating the balanced output of BMP-SMAD1/5 signaling. To address this in the context of *in vivo* heart development, we performed IFM analysis on the localization of p-SMAD1/5 in sections from embryonic mice at E12.5. In WT embryos we observed that SMAD1/5 is phosphorylated predominantly at the base of primary cilia in most cell types of the developing heart, including cells located in the atrial wall, the ventricular trabeculae and the endocardial cushions (Fig. 5I). We also noticed that the ciliary p-SMAD1/5 levels varied between cells in different heart regions as well as between neighboring cells within the same tissue as presented in Fig. 5I. These results suggest that SMAD1/5 phosphorylation at the cilium is highly dynamic during heart development, and we speculate that the variation in ciliary p-SMAD1/5 levels in part could be associated with differential expression of SMURF1. However, this could not be investigated in the present work, since our SMURF1 antibodies did not work well in the mouse tissues. Finally, to address the role of SMURF1 in regulating the level of SMAD1/5 phosphorylation at the cilia in E12.5 mouse embryos, we performed IFM analysis to compare p-SMAD1/5 levels in WT and *Smurf1*^−/−^ tissues with focus on cells in the area surrounding the pharyngeal arch arteries at the aortic sac, which in human embryonic hearts of the same developmental stage shows a very prominent level of SMURF1 expression (Fig. 1D and E). Our analysis showed that phosphorylated SMAD1/5 localizes to the base of cilia in both WT and *Smurf1*^−/−^embryos, but that level of p-SMAD1/5 appeared significantly higher in the mutant embryos (Fig. 5J,K). These results could suggest that SMURF1 operates, at least in part, at the primary cilium to regulate BMP-SMAD1/5 signaling during development of the heart.

## Discussion

The spatiotemporal expression of SMURF1 during human heart development suggests that SMURF1 plays a role in multiple aspects of heart development, including OFT septation and formation of SMC in great and coronary arteries. The observed expression of SMURF1 in many different cell types and in different subcellular compartments further indicates multiple roles of the protein during embryonic heart development. The studies in P19.CL6 cells and mESCs suggest that SMURF1 regulates cell-type specification during cardiomyogenesis possibly through the regulation of ciliary signaling events.

Cardiac septation is a complex process that requires strict regulation of signaling pathways, including TGFβ/BMP signaling^51,52^. The expression pattern of SMURF1 in human embryonic hearts suggests that SMURF1 is involved in OFT septation. In SMURF1-dependent EMT/EndoMT, TGFβ or BMP receptors reside at tight junctions in polarized epithelial/endothelial cells. The binding of ligand ties the receptor II (RII) kinase to the RI:PAR6 complex allowing for RII-mediated phosphorylation of PAR6 and recruitment of SMURF1 to the cell membrane. The complex of PAR6 and SMURF1 targets RHOA for degradation resulting in dissolution of tight junctions and downstream EMT/EndoMT^17,18,20^. At 35 and 38 dpf, SMURF1 is localized to the membrane in OFT mesenchymal cells, which is in agreement with a general role of SMURF1 in regulating EMT/EndoMT and migration. Cardiac fibroblasts evolve from EMT processes during development^53^. In our mESC experiments, depletion of *Smurf1* results in decreased expression of the cardiac fibroblast marker S100A4 (Fig. 5)^54,55^. This reduction in cardiac fibroblasts might partly explain the delay in OFT septation we observed in *Smurf1*^−/−^ embryos, although this requires further investigation.

BMP signaling has roles in addition to the induction of EndoMT during OFT septation, and BMP4 has been found to regulate proliferation and apoptosis in OFT cushions^56^. The ubiquitous expression of SMURF1 in the myocardial cells of the OFT suggest that SMURF1 may regulate the steady state levels of BMP and TGFβ signaling components during OFT septation. SMURF1 is also expressed in the CNC-derived condensed mesenchyme in the OFT, as well as in the distal part of the OFT, suggesting that SMURF1 has a function in CNC-mediated OFT septation. In support of this, disruption of Smurf1 function in *Xenopus* embryos results in perturbed expression of the CNC marker *slug*^14^, SMURF proteins are important for CE movements in mice^13^, and *Smurf1* is expressed in pharyngeal arch four in *Xenopus* embryos^9,15^. Furthermore, SMURF1 is important for cell migration and EMT^17,18,20^, processes that are important during CNC delamination and migration^57^. Whether SMURF1 has a function during CNC delamination and migration, however, requires further investigation. The localization of SMURF1 in the OFT of human embryos, nonetheless, prompted us to investigate if *Smurf1*^−/−^ mouse embryos had defects in the OFT. We found that *Smurf1*^−/−^ mouse embryos at developmental stage E12.5 display delayed septation of the OFT. The *Smurf1*^−/−^ mice were in general smaller than their WT littermates, reminiscent of what was found in *Xenopus* morphants embryos^11^. *Smurf1*^−/−^ mice are on the same somite stage as their WT littermates during development^13^, so the delay in OFT septation is not an indication of an overall developmental delay.

After OFT septation, SMURF1 is expressed in the SMCs of the coronary and great arteries. This could indicate that SMURF1 is important for SMC differentiation. In agreement with this, low levels of BMP-dependent SMAD signaling is required during SMC proliferation^58^. SMURF1 is suggested to be involved in derivation of SMCs from the epicardium together with RHOA, PAR6 and TGFβ-RIII^18^, and *Smurf1* is expressed in SMCs in rats^59^. Furthermore, SMURF1 has been shown to be involved in development of idiopathic pulmonary arterial hypertension (IPAH)^59–61^. Thus, multiple lines of evidence support a role of SMURF1 during SMC differentiation. In line with this, we observed a strong expression of SMURF1 in SMC of the coronary veins and great arteries. At 70 dpf SMURF1 expression is lost in the great arteries as well as in the coronary vessels, which suggests that SMURF1 is important only during differentiation of SMCs. This is in agreement with a requirement of a high level of BMP signaling when the SMC layer has formed to inhibit further proliferation of SMC. We observed a significant reduction of the SMC layer of the aorta in *Smurf1*^−/−^ mice (Fig. 2C,D), supporting a role of SMURF1 during SMC differentiation and/or proliferation. We note that the patient previously reported by Erdogan *et al*., carrying a 7p22 *de novo* duplication encompassing *SMURF1*, presented with coarctation of the aorta^21^. It is tempting to speculate that an increase in SMURF1 dosage may have caused dysregulation of SMC differentiation and/or proliferation during aortic development in this patient.

During the initial phases of heart development, SMURF1 is expressed in the myocardium, which inspired us to investigate the function of SMURF1 during cardiomyogenesis. We used the P19.CL6 cell line and mESC from FHF and SHF lineages. We utilized the CRISPR-Cas9 method to knock-out *Smurf1* in P19.CL6 cells. We differentiated the cells and showed that SMURF1 is a negative regulator of cardiomyogenesis. In the P19.CL6 cells, the activation of BMP signaling is vital for DMSO-induced cardiomyocyte differentiation^62–64^. As far as we are aware, however, the regulation of BMP signaling during differentiation has yet to be investigated. Using WB analysis, we found that the level of SMAD1/5 phosphorylation peaks around day 3 of differentiation, after which it declines. In the KO cells, this level was additionally increased compared to the WT cells. Following day 3, however, no apparent difference was observed, suggesting that SMURF1 regulates the level of SMAD1/5 phosphorylation in the initial phase of differentiation.

To support the data obtained from the P19.CL6 cells, we made a transient knockdown of *Smurf1* in FHF and SHF cardiac progenitor cells followed by cardiac differentiation. The data show that, in SHF-derived cardiomyocytes, a knockdown of *Smurf1* results in increased cardiomyogenesis, reminiscent of what we observed in the P19.CL6 cells. We did not, however, observe any significant effect from *Smurf1* depletion on differentiation of FHF-derived cardiomyocytes. When we analyzed markers for different cell lineages, we observed that SMC and cardiac fibroblast markers were downregulated in SHF cells when *Smurf1* was transiently depleted. These results suggest that SMURF1 regulates BMP signaling during cardiomyogenesis and that SMURF1 is important for cell-type specification.

Multiple signaling pathways that are regulated by SMURF1 play a critical role in heart development, including TGFβ/BMP signaling^33^. These pathways have previously been shown to be coordinated by the primary cilium to promote the differentiation of P19.CL6 cells into cardiomyocytes. Cardiomyogenesis is repressed in cells when the TGFβ pathway is blocked, or when formation of primary cilia is inhibited^34,40^. SMAD1/5 activation was increased in KO cells compared to WT cells at day 3 of differentiation. Further, to delineate how SMURF1 regulates the BMP pathway, we applied BMP2 ligand to pluripotent cells, and to cells differentiated to day 3. We monitored pathway activation by the level of p-SMAD1/5. As expected, *Smurf1* KO induced an increase in p-SMAD1/5 in both pluripotent and differentiated cells, which is in agreement with results obtained in other cell types^9,23,35,36^. To investigate whether SMURF1 functions at the primary cilium, we used cultures of RPE cells to show that SMURF1 as well as BMP receptors localize to the cilium and to the ciliary base region (Fig. 5D,E), which we previously showed plays a critical role in receptor-mediated endocytosis and SMAD2/3-dependent pathway activation^40^. In addition, siRNA-mediated depletion of SMURF1 in RPE cells led to an increased level of p-SMAD1/5 at the primary cilium in the absence of exogenously added BMP ligand (Fig. 5H), supporting the conclusion that SMURF1 operates at the cilium to modulate the basic level of SMAD1/5 activation. IFM analysis of sections from E12.5 mouse embryos shows that phosphorylated SMAD1/5 is predominantly located at the base of the primary cilium throughout the heart. In *Smurf1*^−/−^ embryos we observed significant increase of SMAD1/5 phosphorylation at the pharyngeal arch arteries, a region where SMURF1 is strongly expressed at this stage in human embryos. Thus, our results suggest that SMURF1 regulates BMP signaling in the developing heart. However, these results should be interpreted with some caution. First of all, we observed differences in the developmental stage of the outflow tract between mutant and WT mice, thus the tissue we were comparing may have been at different stages of development. Secondly, the location of the pharyngeal arch arteries was determined from DAPI stained sections which may be imprecise. Third, we were not able to determine expression of SMURF1 in the mouse sections due to lack of functional antibodies. Nevertheless, these are the first data to show that SMURF1 localizes to the primary cilium and affects the level of p-Smad1/5 at the cilium *in vitro* and *in vivo*.

In summary, our study provides evidence that SMURF1 participates in development of the cardiac myocardium, OFT, and blood vessels, in part by regulation of BMP signaling at the primary cilium. Future studies should focus on the role of SMURF1 during CNC migration, and how SMURF1 contributes to the regulation of the balanced and temporal activation and/or deactivation of cellular signaling events at the primary cilium during heart development.

## Methods

### Tissue samples

Human embryonic and fetal heart tissue samples were collected from spontaneous and legal induced abortions. Informed consent was obtained from all contributing women following oral and written information, in accordance with the Helsinki declaration II, and approved by the Research Ethics Committee of the Capital Region (KF–V.100.1735/90). The age of the embryo was based on measurement of crown-rump length. Following dissection, the samples intended for RNA extraction were frozen in liquid nitrogen or treated with RNAlater (ThermoFisher Scientific, Waltham, USA) according to the manufacturer’s instructions. Mouse embryos were collected at E12.5 and E15.5 and fixed in Bouin’s fixatives or 4% paraformaldehyde. The Smurf1 mouse line was generated in Jeff Wrana’s lab. Animal experiments were performed following the recommendations of the Canadian Council on Animal Care. All experimental protocols have been approved by the Animal Care Committee of The Toronto Centre for Phenogenomics, Toronto, Canada.

### Immunohistochemistry and quantification of fluorescence

Tissue samples were dissected into appropriate tissue blocks and fixed for 12–24 hours at 4 °C in 10% neutral buffered formalin, 4% Formol-Calcium, or Lillie’s or Bouin’s fixatives. The specimens were dehydrated with graded alcohols, cleared in xylene, and embedded in paraffin. Serial sections, 3–5 μm thick, were cut in transverse, sagittal or horizontal planes and placed on silanized slides. Sections were deparaffinized and rehydrated in xylene followed by a series of graded alcohol in accordance with established procedures and representative sections were stained with hematoxylin and eosin (HE). For bright field immunohistochemistry, sections were treated with 0.5% H_2_O_2_ for 15 minutes, blocked in 10% goat serum and incubated with primary antibodies followed by secondary antibodies (REAL EnVision Detection System, Peroxidase/DAB [Dako, Agilent Santa Clara, USA]). The sections were dehydrated in graded alcohols prior to mounting and slides were scanned using a panoramic desk II scanner from 3DHISTECH (Budapest, Hungary). The images were processed using Panoramic Viewer (3DHISTECH). For immunofluorescence microscopy, sections were deparaffinized as described above, treated with TEG buffer before blocking with Dako REAL^TM^ Antibody Diluent (Agilent, Santa Clara, USA) and left in primary antibodies overnight at 4 °C in a humidified chamber. After PBS washing steps, cells were incubated in corresponding Alexa Flour® secondary antibodies for 45 min at room temperature. Cell nuclei were labeled by DAPI staining. Coverslips were mounted with either Dako fluorescent mounting medium or with PBS containing 90% glycerol, and 2% N-propyl-gallate and subjected to either confocal or epifluorescence microscopy. Laser scanning confocal microscopy images were captured using a Carl Zeiss LSM 780 microscope and analyzed throughout the z-axis of the section. Individual optical sections were stored as TIFF files using Zeiss ZEN Vision v10 (Zeiss, Oberkochen, Germany) and images were processed using ImageJ software^65^. Epifluorescence microscopy images were captured on a fully motorized Olympus BX63 upright microscope with an Olympus DP72 color, 12.8-megapixel, 4.140 × 3.096-resolution camera and with a fully motorized and automated Olympus IX83 Inverted microscope with a Hamamatsu ORCA-Flash 4.0 camera (C11440-22CU). The software used was Olympus CellSens dimension, which was able to do deconvolution on captured z stacks, and images were processed for publication using Adobe Photoshop CS6. For quantification of levels of phospho-SMAD1/5 at the ciliary base, a circular outline was drawn around each base and using the measurement and region of interest (ROI)-function in the CellSens dimension software (Olympus) the Mean Green Fluorescence Intensity was measured in this area.

### Cell cultures

P19.CL6 cells were cultured as previously described^34,40^. For differentiation experiments 4,000 cells/cm^2^ were seeded in Petri dishes with MEM-alpha medium containing 1% DMSO to induce cardiomyogenesis. Differentiating cells were kept at 37 °C, 5% CO_2_ and 95% humidity, and medium was changed every second or third day. For stimulation experiments cells were seeded at a density of 17,000 cells/cm^2^. For overexpression studies 2 × 10^6^ cells were electroporated with 1 ng plasmid using the Nucleofector^TM^ 2b device from Axama (Lonza, Basel Switzerland) as previously described^66^. After electroporation cells were seeded at a density of 4,500 cells/cm^2^ to account for the cell death caused by transfection. HEK-293 cells were cultured in DMEM 1965 medium supplemented with NaHCO_3_, Hepes, glutamax, antibiotics and FBS, and kept under incubator conditions as described above. The cells were passaged every second day. Immortalized human retinal pigment epithelial (hTERT-RPE1/RPE) cells were cultured to 80% confluency and then serum depleted for 24 hours to induce growth arrest and formation of primary cilia as previously described^48^. Mouse Embryonic Stem (mES) Cells^Isl1−Cre; Ai9; Nkx2.5−GFP^ were derived from mice harboring *Isl1Cre*; *Ai*9; *Nkx2*.*5*-*GFP*. ES cells were maintained on gelatin-coated dishes in maintenance medium (Glasgow minimum essential medium with 10% fetal bovine serum and 1000 U/ml ESGRO (Millipore, Billerica, MA), Glutamax, sodium pyruvate and MEM non-essential amino acids (Life Technologies). Cardiac progenitor cells resembling heart field derivatives were induced with Activin A, BMP4, and VEGF (R&D Systems, Minneapolis, MN). After 5 days of differentiation, SHF and FHF cells were purified by Fluorescence-Activated Cell Sorting (FACS) (SH800, Sony Biotechnologies) based on their expression of GFP and/or RFP^47^.

### Plasmids and oligonucleotides

For CRISPR-Cas9 genome editing a pCas9-GFP vector (hCas9, 9553 bp, plasmid 44719) and an empty sgRNA vector (gRNA_Cloning Vector, 3915 bp, plasmid 41824) were obtained from Addgene (Cambridge, MA, USA). Two sgRNAs for deletion of the Smurf1 gene were designed using CRISPR DESIGN (http://crispr.mit.edu/), and each sgRNA was cloned into the sgRNA vector using the protocol developed by Mali *et al*.^67^. Shortly, the sgRNAs were synthesized as 60-mer oligonucleotides (Suppl. Table 2) (Integrated DNA technologies, Coralville, IA). Double stranded 100 bp DNA fragments were generated using a Phusion® High-Fidelity DNA Polymerase kit [1 rxn: 10 μl Phusion reaction buffer (HF), 2.5 μl dNTP (10 mM), 2 μl Smurf1_gRNA#1 F, and 2 μl Smurf1_gRNA#1 R, 2 μl Phusion DNA Polymerase, 31.5 μl ddH_2_O]. The double stranded fragment was ligated into the sgRNA empty vector using the Gibson Assembly® Master Mix [5 μl (2×) Gibson assembly mix, 2 μl gRNA empty vector (linearized by *AflII*), 1 μl 100 bp PCR fragment, 2 μl ddH_2_O] and incubated at 50 °C (60 min). For overexpression of Smurf1, full length *Smurf1* mouse cDNA cloned into pCMV-SPORT6 and was purchased from ImaGenes GmbH (now Source Bioscience, Nottingham, UK). An empty pCMV-SPORT6 vector was used for mock transfection. For transfection of RPE cells SMURF1 siRNA was obtained from Ambion (ID: s32796) (ThermoFisher Scientific).

### Generation of mutant P19.CL6 cells

P19.CL6 cells were transfected with the two sgRNA-plasmids and the GFP-plasmid (6 ng each). After two days, the cells were sorted by FACS, and top 5% of GFP-positive cells was selected and transferred to MEM-alpha medium with serum and antibiotics. GFP-positive cells were seeded at a concentration of ~50 cells per ml to allow selection of single clones. DNA was isolated and amplified from a total of 66 clones, PCR products were separated using electrophoresis on a 2% agarose gel. Analysis of PCR products spanning the site of mutation suggested that 32 out of 66 cell clones (48%) were homozygous for the mutation, 23 (35%) were heterozygous and 11 (17%) were homozygous for the normal *Smurf1* allele (Suppl. Fig. 4B,C). Clones with a homozygous deletion or no deletion were selected for further analysis. Selected clones were genotyped by Sanger sequencing using primers spanning the CRISPR-Cas9 target site in *Smurf1*. Excess dNTPs and primers were removed before sequencing with exonuclease I (USB) and Shrimp alkaline phosphatase. Sequences were analyzed using CromasLite software edition 2.1.1 (Technelysium Pty Ltd, South Brisbane, Australia).

### Knock-down by siRNA

RPE cells were cultured to 60% confluency prior to transfection with SMURF1 siRNA or scrambled oligonucleotides (Mock) with similar GC content using DharmaFECT Duo (Dharmacon, Cat#T-2010-03) as previously described^68^. Twentyfour hours after transfection the medium was changed to serum deprived medium and the cells were cultured for 24 hours to induce growth arrest and formation of primary cilia. mESC were transfected with siRNA immediately after FACS sorting. Cells were transfected with Lipofectamine LTX or Lipofectamine 2000 (Life Technologies) in single-cell suspensions^47^.

### BMP signaling assays

For BMP2 signaling experiments in un-differentiated P19.CL6 cells, the medium was changed to medium without serum and antibiotics 24 hours prior to stimulation. Cells were stimulated with BMP2 (355-BM-050 R&D biosystems, Oxon, UK) at a final concentration of 200 ng/ml in medium without serum and antibiotics. When stimulating differentiated P19.CL6 cells, the cells were differentiated to day three, and new differentiation medium containing 200 ng/ml BMP2 was added to the cells. For both setups, cells were lysed in 1% SDS lysis-buffer at the indicated time points.

### RNA extraction

Total RNA from human embryonic hearts was isolated using TRIzol Reagent. RNA from P19.CL6 cells was purified using the NucleoSpin RNA II kit (Macherey-Nagel, Düren, Germany) according to manufactures protocol, and stored at −80 °C. RNA samples from human adult hearts were obtained from Stratagene (San Diego USA) and Clontech (Mountain View, USA).

### Real-Time Quantitative Reverse Transcription PCR qRT-PCR

RNA was reverse transcribed with a HT11V primer using SuperscriptII. Quantitative PCR analysis was carried out on an ABI 7500 Fast real-time PCR system using a LightCycler FastStart DNA MasterPLUS SYBR Green kit. All primer sets were designed to span at least one intron. Samples were run in duplicates and normalized to the average expression value of at least two housekeeping genes (for human samples: *GAPDH*, *COX4* and *ATP6* and for P19.CL6 samples: *Psmd4* and *Gapdh*). Data from human samples were analyzed using the standard curve method and data from P19.CL6 cells were analyzed using the 2−ΔΔCT method^69^. Please see Suppl. Table 3 for primer sequences.

### SDS-PAGE, Western Blotting and quantification of band intensities

P19.CL6 cells were lysed in 1% SDS lysis-buffer and homogenized by sonication. Protein concentration was standardized using the BioRad DC Protein Assay. Proteins were resolved by gel electrophoresis on 10% bis-tris gels and run with NuPAGE MOPS SDS running buffer as previously described^70^. Proteins were subsequently electrophoretically transferred to nitrocellulose or PVDF membranes. The membranes were blocked with BSA or Odyssey^®^ Blocking Buffer (Li-COR Biosciences, Lincoln, NE) and incubated with primary antibodies overnight. HRP-conjugated (Dako, Agilent) or IRDye^®^ (Li-COR Biosciences) secondary antibodies were applied and the blots were developed using the ECL^TM^ detection kit and FUSION-FX, or Odyssey^®^ LCx imaging system (Li-COR Biosciences). Please see Suppl. Table 1 for antibody specifications. For western blots (WB) shown in Fig. 3, the relative intensities were normalized to the loading control (p150^glued^) and band intensities were estimated from arbitrary densitometric values obtained with UNSCAN-IT version 5.1 software (Silk Scientific, Inc., Orem, Utah, USA). For WBs shown in Fig. 5, Image Studio lite version 5.2.5 (LICOR, Biosciences) was used to measure the intensities of the bands. All intensities of the loading control (HPRT) bands were divided with the highest HPRT intensity to get a relative normalized value. The band intensities of the protein of interest were divided by the relative HPRT value in their respective lane.

### Immunocytochemistry

Cells were grown on glass coverslips, and fixed in 4% PFA and permeabilized in 2% Triton X-100/PBS. 2% BSA was used for blocking and cells were incubated with primary antibodies followed by incubation with Alexa flour^®^ secondary antibodies (ThermoFisher Scientific). DAPI was used to mark nuclei. Immunofluorescence images were captured on a fully motorized Olympus BX63 upright microscope with a DP72 color, 12.8 megapixel camera and differential interference contrast (DIC). The software used was Olympus CellSens dimension, which is able to do 3D isosurface projections on captured z stacks. Image adjustments were carried out in Adobe Photoshop CS4 version 11.0. Please see Suppl. Table 1 for antibody specifications.

### Flow cytometry

Cells were trypsinated, and 2 × 10^5^ cells were fixed in 2% PFA for 20 min at room temperature. The cells were incubated with 5% goat serum with 0.5% Saponin. Cells were incubated with either α-actinin or control antibody with subsequent incubation with Alexaflour^®^ secondary antibody. Cells were analyzed on a BD Accuri™ Flow Cytometer (BD Biosciences). Data was analyzed using the BD Accuri^TM^ S6 Software (BD Biosciences).

### Data availability statement

Data supporting the conclusions of this article are included in the article and supplementary files.

## Electronic supplementary material

Supplemental Material
