# Supplementary material for: The E3 ubiquitin ligase SMURF1 regulates cell-fate specification and outflow tract septation during mammalian heart development
Source: Sci Rep. 2018 Jun 22;8:9542. doi: 10.1038/s41598-018-27854-8 (PMC6015040; doi:10.1038/s41598-018-27854-8)
Supplement: Supplementary file 1 — Supplemental Material [file 41598_2018_27854_MOESM1_ESM.pdf]

Supplementary Information

The E3 ubiquitin ligase SMURF1 regulates cell-fate specification and outflow tract septation during mammalian heart development

K Koefoed, J Skat-Rørdam, P Andersen, CB Warzecha, M Pye, TA Andersen, KD Ajbro, E Bendsen, M Narimatsu, F Vilhardt, LB Pedersen, JL Wrana, RH Anderson, K Møllgård, ST Christensen and LA Larsen

Suppl. Figure 1

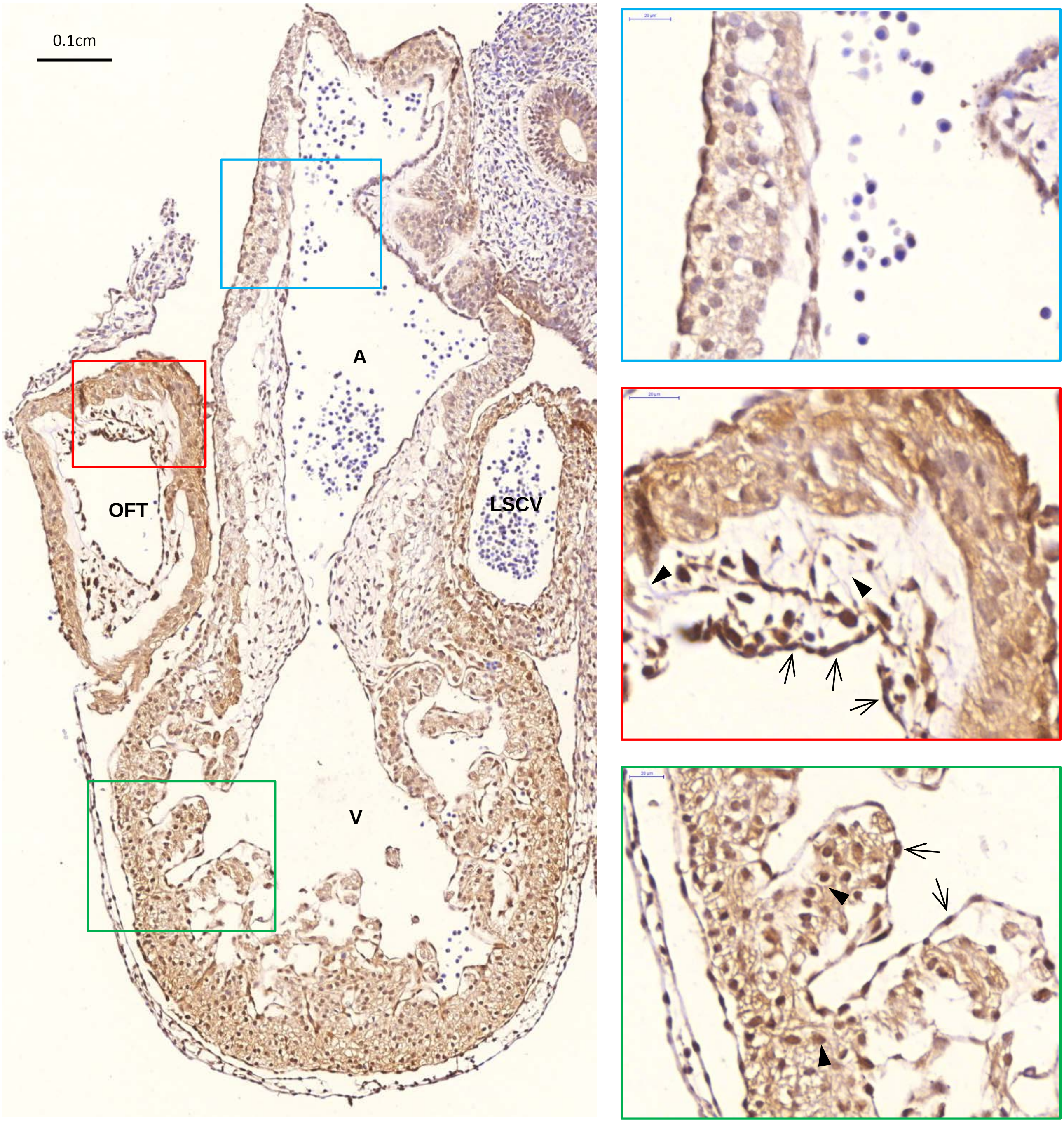

Suppl. Figure 2

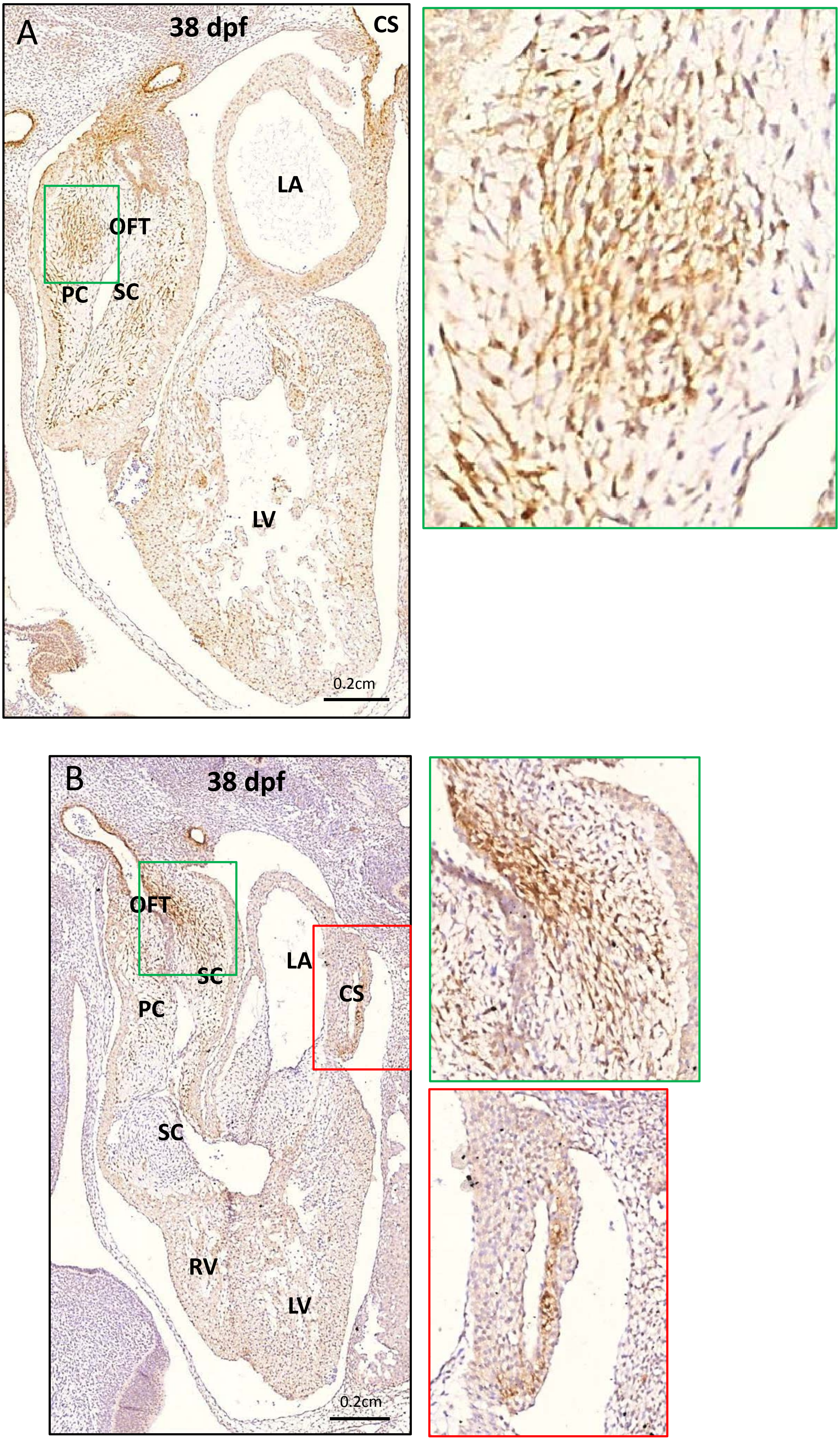

Suppl. Figure 3

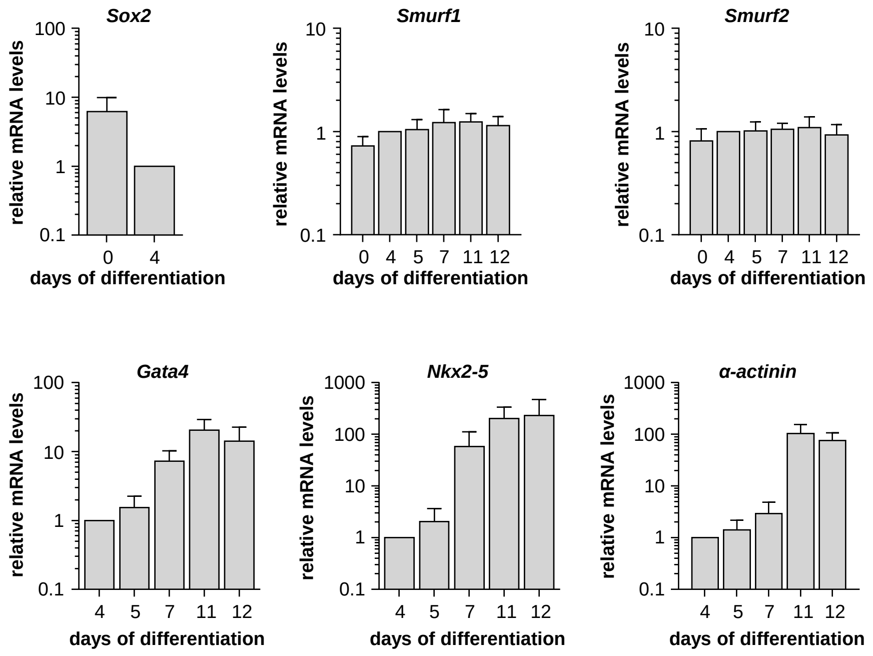

Suppl. Figure 4

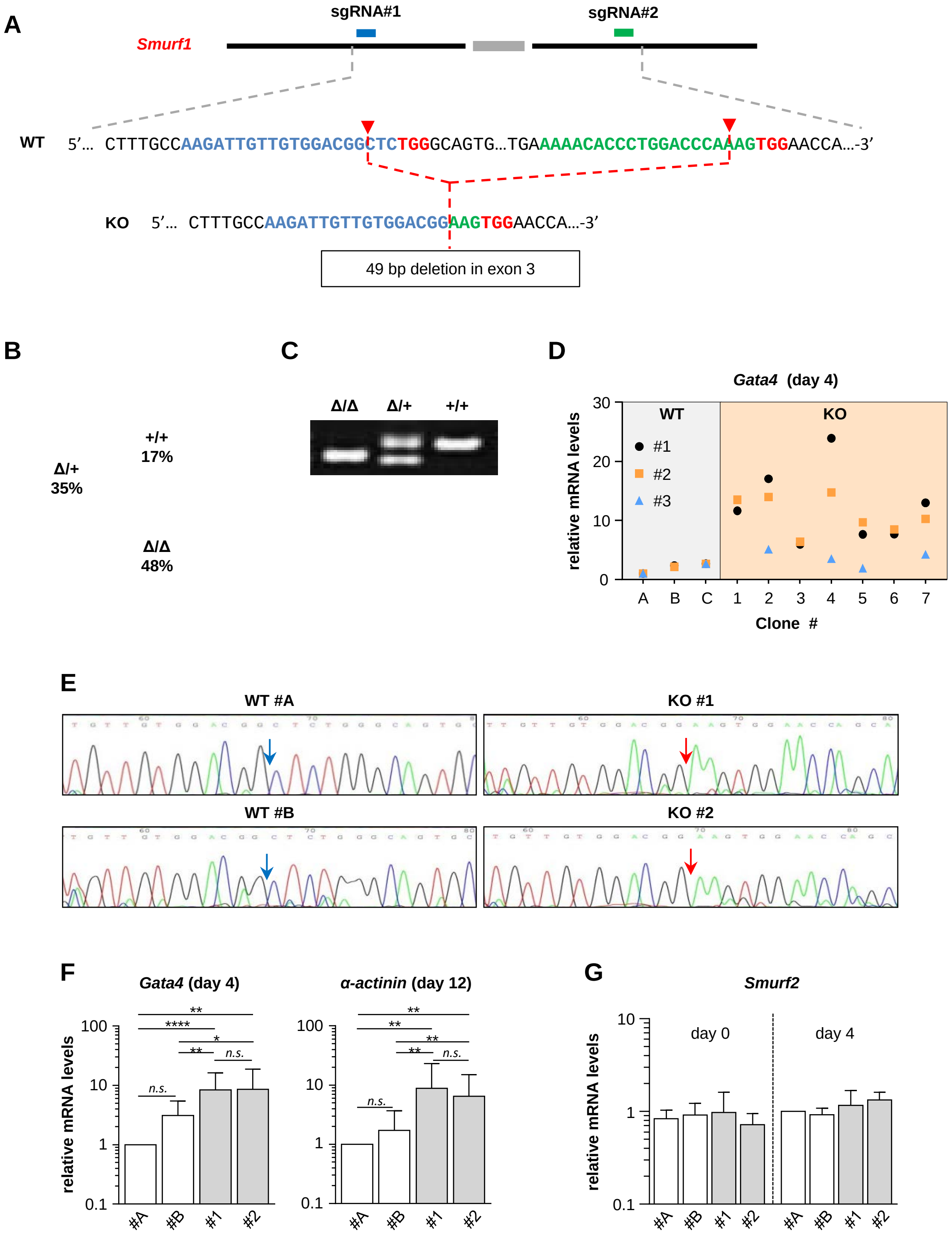

Suppl. Figure 5

Western blot shown in figure 3A

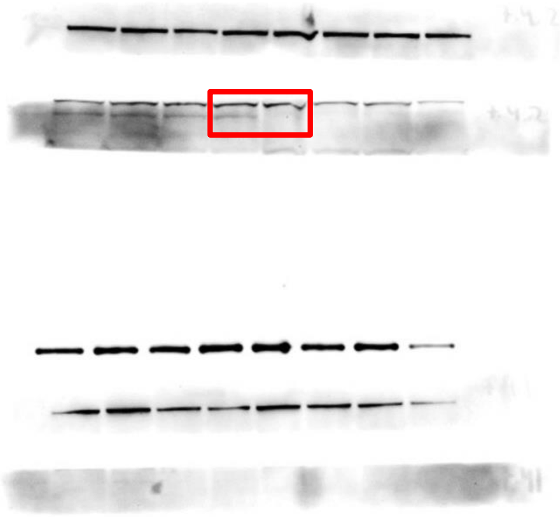

Western blot shown in figure 3D

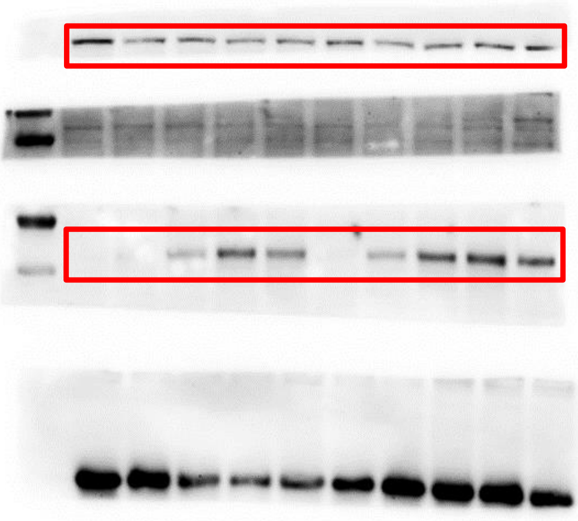

Western blots shown in figure 5A

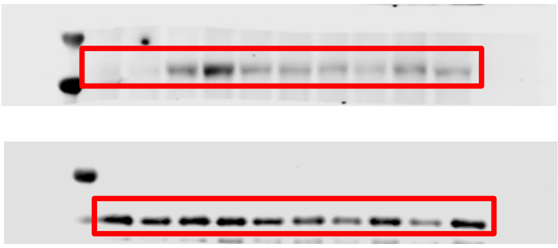

Western blots shown in figure 5B

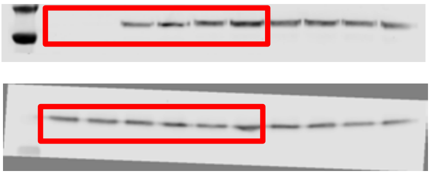

Western blots shown in figure 5C

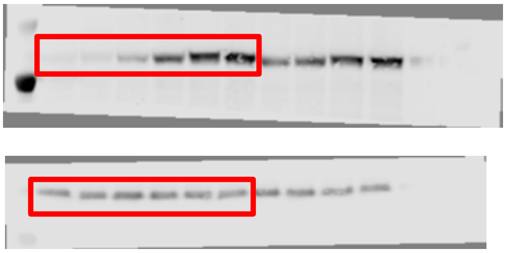

Western blots shown in figure 5F

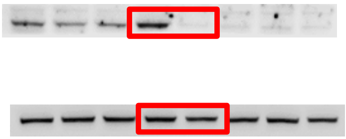

Suppl. Figure 5 cont.

Agarose gel shown in figure 3A and 4S

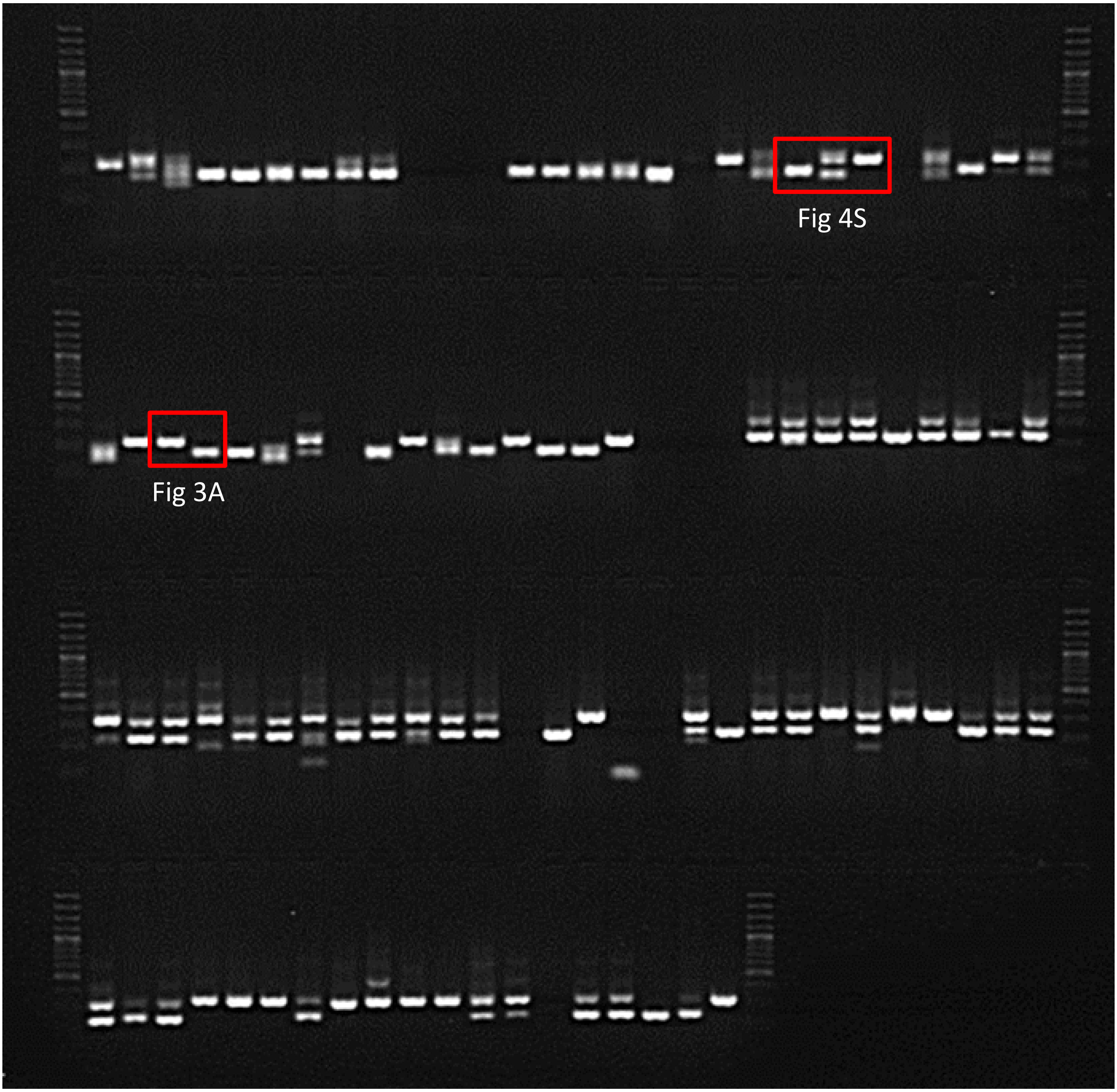

Suppl. Table 1: Primary antibodies

Antigen	abbreviation	Company	Catalog#	Application
alpha-actinin	α-ACTININ	Sigma	A7811	FACS
alpha smooth muscle actin	α-SMA	Sigma	A2547	IHC
acetylated α-tubulin	Ac-Tub	Sigma	T7451	ICC
ADP-ribosylation factor-like 13B	ARL13B	Protein tech	17711-1-AP	IHC
BMP receptor II	BMPRII	Santa cruz	Sc-5682	ICC
BMP receptor I	BMPRI	Santa cruz	Sc-25454	ICC
GATA Binding Protein 4	GATA4	Santa cruz	sc-1237	IFM WB
hypoxanthine-guanine phosphoribosyl transferase	HPRT	Protein tech	15059-1-AP	WB
mouse IgG1 negative control antibody	IgG	Biorad	MCA1209	FACS
$p150^{glued}$	$p150^{glued}$	BD-Biosciences	610474	WB
phospho-SMAD1/5	p-SMAD1/5	Cell signaling	9516S	ICC, WB
phospho-SMAD1/5	p-SMAD1/5	Santa cruz	Sc-12353	IHC
SMURF1	SMURF1	Abcam	Ab57573	IHC, WB (P19.CL6)
SMURF1	SMURF1	Sigma	WH005715aM1	WB (RPE)
SMURF1	SMURF1	Santa cruz	Sc-25510	ICC
T-box transcription factor 2	TBX2	Abcam	Ab33298	IHC

Suppl. Table 2: 60-mer oligonucleotides containing Smurf1 sgRNA#1 and sgRNA#2

Name	Species	Oligonucleotide 5'→3'
**sgRNA#1F**	Mouse	TTTCTTGGCTTTATATATCTTGTGGAAAGGACGAAACACCGAGATTGTTGTGGACGGCTC
**sgRNA#1R**	Mouse	GACTAGCCTTATTTTAACTTGCTATTTCTAGCTCTAAAACGAGCCGTCCACAACAATCTC
**sgRNA#2F**	Mouse	TTTCTTGGCTTTATATATCTTGTGGAAAGGACGAAACACCGAAACACCCTGGACCCAAAG
**sgRNA#2R**	Mouse	GACTAGCCTTATTTTAACTTGCTATTTCTAGCTCTAAAACCTTTGGGTCCAGGGTGTTTC

Suppl. Table 3: Primers

Gene	Annealing temp. (^{o}C)	Forward primer	Reverse primer
**Primers used for qRT-PCR in P19.CL6 cells**			
α-Actinin	60°	CTGGAGTTTGCCAAGAGAGC	AACAGTGCTGTAGGGGTTGC
Gapdh	60°	AACAGCAACTCCCACTCTTC	TGGTCCAGGGTTTCTTACTC
Gata4	60°	CCTCCAGCGGTAACTCC	CTGATTACGCGGTGATTATG
Nkx2.5	60°	CAAGTGCTCTCCTGCTTTCC	GTACCGCTGTTGCTTGAAGC
Psmd4	60°	GCAAGATGGTGTTGGAGAGC	TTTGGGTTGGACAGTGTGG
Smurf1	60°	AGGCTCTGCAAGGCTCTACAG	GGTGGTTGTGAGCAAGACTCTG
Smurf2	50°	AAGAACTACACAGTGGGAACGC	CACGTTGCACCATTTGTTCC
Sox2	60°	GGCAGCTACAGCATGATGCAGGAGC	CTGGTCATGGAGTTGTACTGCAGG
**Primers for qRT-PCR in human hearts**			
ATP6	60°	CTGAATGCTCCACTTTTTCAATTCT	CGACAGCGATTTCTAGGATAGTCAG
COX4	60°	TGAGATGAACAGGGGCTCGAAC	TTCGTAGTCCCACTTGGAGGCTAAG
GAPDH	60°	GGAAGGTGAAGGTCGGAGTCAA	GATCTCGCTCCTGGAAGATGGT
SMURF1	60°	AGCATGAACTGAAACCCAATGG	GCTCATTGAACCCCTTCTGC
**Primers used for genotyping**			
Smurf1	60°	GCTGTGCCACCAGGTAGACT	GCCTGACAGCCACTTTTCC

Supplemental Figure legends

Suppl. Figure 1: Expression of SMURF1 in the 35 dpf heart

IHC staining of SMURF1 in human heart section from 35 dpf. Blue box shows SMURF1 expression in atrial myocardium, red box shows SMURF1 expression in the OFT. Arrow heads mark endocardial cells, open arrows mark mesenchymal cells. Green box shows SMURF1 expression in the ventricle. Abbreviations: A: Atrium, LSCV: Left superior caval vein, OFT: Outflow tract, V: Ventricle.

Suppl. Figure 2: Expression of SMURF1 in the 38 dpf heart

A: IHC staining of SMURF1 in human heart section from 38 dpf. **B**: IHC staining of SMURF1 in a neighboring section to A. Abbreviations: CS: coronary sinus, LA: Left atrium, LV: Left ventricle, OFT: Outflow tract, PC: Parietal cushion, RV: Right ventricle, SC: septal cushion.

Suppl. Figure 3: Differentiation of P19.CL6 cells

Quantitative RT-PCR analysis of the relative expression of *Sox2, Gata4, Nkx2-5, α-actinin, Smurf1* and *Smurf2* during differentiation of WT P19.CL6 cells. Data are normalized to *Gapdh* and *Psmd4*, n=8.

Suppl. Figure 4: Validation of CRISPR clones

A: Schematic illustration of generation of *Smurf1* KO clones. The two sgRNAs (sgRNA#1 and sgRNA#2) were designed to target exon three in the *Smurf1* gene. The Cas9 cuts (red arrow) a fragment of 49bp in *Smurf1* that was predicted to produce a premature stop codon and a truncated gene product (sgRNA#1, blue; sgRNA#2, green). **B:** Pie-chart showing the distribution of WT heterozygous and KO clones. Δ/Δ: knockout, Δ/+: heterozygous, +/+: WT. **C:** Agarose gel of the PCR product following CRISPR-Cas9 treatment. **D:** qRT-PCR analysis *Gata4* mRNA expression on day 4 of differentiation of three WT clones #A-C and seven KO clones #1-7. Data is normalized to *Gapdh* and *Psmd4.* Three different experiments are plotted (#1-3). **E:** Results from Sanger sequencing of two WT (#A and #B) and two knockout clones (#1 and #2). Blue arrow: no Cas9 activity and WT sequence, red arrow: Cas9 activity and 49bp deletion. **F:** qRT-PCR analysis of two WT and two KO clones. The relative mRNA levels of *Gata4* and *α-actinin* after 4 and 12 days of DMSO stimulation, respectively, were analyzed. Data are normalized to *Gapdh* and *Psmd4*. Ratio-paired t-test was used for statistical analysis *$p<0.05$ **$p<0.01$****$p<0.0001$, n=10 for *Gata4* and n=8 for *α-actinin*. **G:** qRT-PCR analysis of two WT and two KO clones. The relative mRNA levels of *Smurf2* on days 0 and 4 of DMSO stimulation were analyzed. Data are normalized to *Gapdh* and *Psmd4.* Original picture of the agarose gel is shown in supplemental figure 5.

Suppl. Figure 5: Original pictures of western blots and agarose gels

The pictures taken of the original blots and gels before cropping of the pictures is shown. The part of the pictures used in figure 3, 5 and S4 is marked with red box.
